# Effects of exogenous proteases without or with carbohydrases on nutrient digestibility and disappearance of non-starch polysaccharides in broiler chickens

**DOI:** 10.3382/ps/pev260

**Published:** 2015-10-23

**Authors:** O. A. Olukosi, L. A. Beeson, K. Englyst, L. F. Romero

**Affiliations:** * Monogastric Science Research Centre, Scotland's Rural College, Edinburgh, EH9 3JG, UK; † Englyst Carbohydrates Ltd, Southampton, Hampshire, SO16 7NP, UK; ‡ Dupont Industrial Biosciences, Marlborough, SN8 1XN, UK

**Keywords:** broilers, carbohydrase, component sugars, non-starch polysaccharides, protease

## Abstract

The objective of the current study was to evaluate the effect of a subtilisin protease, without or with inclusion of carbohydrases, on digestibility and retention of energy and protein, as well as the solubilization and disappearance of non-starch polysaccharides (NSP) from corn-soybean meal based diets fed to broiler chickens. Two hundred eighty-eight Ross 308 male broiler chickens were used for the experiment. On d 14, the birds were weighed and allocated to 6 treatments and 8 replicates per treatment with 6 birds per replicate. Treatments were: 1) corn-soybean meal based control diet; 2) control diet plus supplemental protease at 5,000 (P5000) protease units (PU)/kg); 3) control plus 10,000 PU/kg protease (P10000); or control plus an enzyme combination containing xylanase, amylase, and protease (XAP) added to achieve protease activity of: 4) 2,500 PU/kg (XAP2500); 5) 5,000 PU/kg (XAP5000); or 6) 10,000 PU/kg (XAP10000). The enzymes in XAP were combined at fixed ratios of 10:1:25 of xylanase:amylase:protease. Data were analyzed by ANOVA and specific orthogonal contrasts between treatments were performed. Addition of xylanase and amylase increased (*P* < 0.05) the ileal digestibility of protein by 4.2% and 2.1% at XAP5000 and XAP10000, respectively (relative to P5000 and P10000, respectively), exhibiting a plateau after the XAP5000 dose. Increment (*P* < 0.05) in AME due to protease was evident, particularly in P10000. At the ileal level, XAP reduced (*P* < 0.05) the flow of insoluble xylose and arabinose, which indicates an increase in the solubilization of arabinoxylan polymers in the small intestine. Protease on its own reduced (*P* < 0.05) the flow of insoluble arabinose but did not affect the flow of insoluble xylose. XAP reduced (*P* < 0.05) the pre-cecal flow of insoluble and total glucose and galactose. It was concluded that whereas protease by itself improved nutrient utilization and increased solubilization of NSP components, at the lower dose, a combination of xylanase, amylase, and protease produced effects greater than those of protease alone.

## INTRODUCTION

The use of exogenous proteases in poultry diets has gained momentum during the last decade. The first commercial protease was introduced into the poultry feed market in the 1990s in combination with other enzymes, with the aim to increase the energy and protein digestibility of grain and oilseed meal based diets (Simbaya et al., 1996). A number of proteases are now available commercially and their use has significantly increased as a result of significant pressure on the price of soybean meal, which has motivated nutritionists to further evaluate proteases for their ability to improve protein and amino acid digestibility of diets. Several studies have documented increments in the ileal digestibility of protein and amino acids of diets fed to broiler chickens with protease supplementation (Romero et al., 2013, 2014). The use of protein and amino acid matrices for protease containing products in diet formulation is becoming common practice. Nonetheless, the understanding of the mode of action of proteases in the gastrointestinal tract of chickens is still limited.

Although the link between exogenous protease activity and digestion of dietary proteins is obvious, the intestinal system is complex and the effects of proteases cannot be limited to hydrolysis of dietary proteins only. Interactions between the digestions of other nutrients in the feed matrix are possible, as well as changes in the microbial communities due to modifications in the availability of easily accessible proteins in different parts of intestinal lumen (Morita et al., 1998; Scott et al., 2013). Additionally, mild interactions with the intestinal mucosa such as an increment in the thickness of the mucus layer in the intestinal lining of young chickens (Peek et al., 2009) have been reported, and ascribed potentially beneficial effects in conditions of coccidiosis challenges, which have not been fully demonstrated.

Proteins are an important part of the structure of cell walls of vegetable ingredients, where they are embedded in complex matrices with carbohydrates (Parker et al., 1999). Research in ruminant in-vitro and in-vivo models has demonstrated the ability of exogenous subtilisin proteases to increase the solubilization and fermentation of hemicellulose in grains and alfalfa (Colombatto and Beauchemin, 2009). The authors postulated that these enzymes acted by removing structural proteins in the cell wall, allowing faster access of ruminal microbes to digestible substrates. To our knowledge, the effect of exogenous proteases on the disappearance of non-starch polysaccharides (**NSP**) has not been tested in commercially relevant diets in broiler chickens.

The objective of the current study was to evaluate the effect of a subtilisin protease, without or with inclusion of carbohydrases, on ileal digestibility and total tract retention of energy and protein, as well as the solubilization and disappearance of NSP from corn-soybean meal based diets fed to broiler chickens.

## MATERIALS AND METHODS

All the animal experimentation procedures were approved by Scotland's Rural College's Animal Experiment Committee.

### Birds, Diets and Sample Collection

Two hundred eighty-eight Ross 308 male broiler chickens at 14 d old were used for the experiment. The birds were raised together in a group and received a standard diet that met the nutrient and energy recommendations for the breed (Ross, 2007). On d 14, the birds were weighed and allocated to 48 metabolism cages which consisted of 6 treatments and 8 replicate cages per treatment with 6 birds per replicate cage.

The 6 treatments were a corn-soybean meal based control diet that met the nutrient specifications for Ross 308 broilers but was marginally low in energy (95%) and the control diet plus supplemental protease at 5,000 (P5000), or 10,000 (P10000) protease (**P**) units/kg, or a commercial enzyme combination (Axtra XAP; Danisco Animal Nutrition, DuPont Industrial Biosciences, Marlborough, UK) with fixed ratios (10:1:25) of xylanase (2,000 xylanase units/kg), amylase (200 amylase units/kg), and protease (5,000 protease units/kg; XAP) which was dosed to supply protease activities at 2,500 (XAP2500; 50% XAP); 5,000 (XAP5000; 100% XAP); or 10,000 (XAP10000; 200% XAP) protease units/kg.

The xylanase originated from *Trichoderma reesei*; the amylase originated from *Bacillus licheniformis*; and the protease originated from *Bacillus subtilis*. One xylanase unit was defined as the amount of enzyme that releases 0.48 μmol of reducing sugar as xylose from wheat arabinoxylan per minute at pH 4.2 and 50°C. One unit of *Bacillus licheniformis* α-amylase was the amount of enzyme required to release, in the presence of excess α-glucosidase, 0.20 μmol of glucosidic linkages expressed as p-nitrophenol equivalents from a maltoheptaoside substrate per minute at pH 8.0 and 40°C. One protease unit (**PU**) was defined as the amount of enzyme that releases 1.0 μg of phenolic compound, expressed as tyrosine equivalents, from a casein substrate per minute at pH 7.5 and 40°C.

Diets were manufactured in one batch, which was subdivided into 6 experimental diets, 5 of them containing enzymes. Concentrates of the test enzymes were sprayed into a wheat carrier, activity was measured and standardized, and the enzyme preparations were added to the respective diets in dry form at 0.5 g/kg after being premixed with 5 g of maize or wheat/kg of the diets. Titanium dioxide (0.5%) was added to all the diets as an indigestible marker. The ingredients and chemical composition of the diets are presented in Table 1. Excreta were collected from the birds on d 19 and 20 whereas ileal digesta were collected on d 21 following euthanasia of the birds by an overdose of sodium pentobarbital. Contents of the lower half of the ileum were obtained by gentle flushing with distilled water. The ileum was defined as the portion of the small intestine extending from the Meckel's diverticulum to a point approximately 40 mm proximal to the ileocecal junction (Ravindran et al., 2005). Digesta from birds within a cage were pooled, resulting in 8 samples per dietary treatment. The digesta samples were frozen immediately after collection, lyophilized, and processed.

**Table 1.** Ingredient and chemical compositions of the experimental control diet.

Ingredients	g/kg
Corn	501.1
Corn DDGS	100.0
Soyabean meal	288.3
Canola Meal	50.0
Soybean oil	21.6
L-Lysine·HCl	3.3
DL-methionine	2.8
L-threonine	1.2
Titanium dioxide	3.0
Salt	3.3
Limestone	10.9
Dicalcium Phosphate	11.5
Poultry vitamin-mineral premix^1^	3.0
Total	1000
Analyzed nutrient composition, g/kg	
Protein	210
Gross energy, kcal/kg	4,043
Ca	10.9
Total P	7.03
Starch	350.0
Total sugar (glucose + sucrose)	7.22
Resistant galactose	12.59
Resistant glucose	6.66
Total resistant oligosaccharide	22.97
Soluble NSP^2^	24.7
Insoluble NSP	97.7
Total NSP	122.4

^1^The premix provided (units kg^−1^ diets): Vit A 16,000 iu; Vit D_3_ 3,000 iu; Vit E 75 iu; Vit B_1_ 3 mg; Vit B_2_ 10 mg; Vit B_6_ 3 mg; Vit B_12_ 15 μg; Vit K_3_ 5 mg; Nicotinic acid 60 mg; Pantothenic acid 14.5 mg; Folic acid 1.5 mg; Biotin 275 μg; Choline chloride 250 mg; Iron 20 mg; Copper 10 mg; Manganese 100 mg; Cobalt 1 mg; Zinc 82 mg; Iodine 1 mg; Selenium 0.2 mg; Molybdenum 0.5 mg.

^2^NSP – non starch polysaccharides.

### Chemical Analysis

Chemical analyses were done according to AOAC (2006) methods unless otherwise indicated. Diets, ileal digesta, and excreta were analyzed for Ti, DM, N, ether extract, minerals, GE, NSP components, and resistant oligosaccharides (**RO**). Dry matter was determined by drying the samples in a drying oven (Uniterm, Russel-Lindsey Enginering Ltd., Birmingham, England, UK) at 105°C for 24 hours (Method 934.01). Total N content was determined by the combustion method (Method 968.06) whereas ether extract was determined in a soxhlet extractor (Method 922.06). Gross energy value was determined in an adiabatic bomb calorimeter (Model 6200, Parr Instruments, Moline, IL) using benzoic acid as an internal standard. Titanium concentration in the samples was determined using the method of Short et al. (1996). Mineral content was determined using Inductively Coupled Plasma – Optical Emission Spectroscopy (Method 990.08) following digestion, in turn, in concentrated HNO_3_ and HCl. Analyses for the sugar components of NSP and for RO were done using the methods of Englyst et al. (1994).

### Calculations and Statistical Analysis

Digestibility and nutrient retention values were calculated using the index method as described previously (Olukosi et al., 2007). Calculations of the flow of NSP components were done using the concentrations of Ti in both the diet and the digesta or excreta as well as the concentration of the NSP component in digesta or excreta. All the data were analyzed using the mixed model procedure of SAS 9.2 (diets are fixed effects and the blocks are random effects) as appropriate for a Randomized Complete Block Design with pens as the experimental unit. Significantly different means were separated by orthogonal contrast tests.

## RESULTS

The expected nutrient content of the experimental diets were met, however analyzed protease activity for XAP2500 was approximately 50% of the expected value. The analyzed PU/kg were 3,687 (for treatment P5000), 9,851 (for treatment P10000), 1,243 (for treatment XAP2500), 5,515 (for treatment XAP5000), and 10,203 (for treatment XAP10000). The anticipated activities were 2,500 PU/kg for treatment XAP2500; 5,000 PU/kg for treatments P5000 and XAP5000; and 10,000 PU/kg for treatments P10000 and XAP10000.

The effect of the treatments on ileal digestibility and total tract nutrient retention are shown in Table 2. There were significant diet effects (*P* < 0.01) for all the nutrients and gross energy utilization at both the ileal and total tract level, except for fat digestibility at the ileal level. Supplementation of protease or XAP improved (*P* < 0.01) DM and N utilization relative to the control at both the ileal and total tract levels. Ileal and total tract DM and N utilization were greater (*P* < 0.01) in XAP5000 compared with P5000. Gross energy retention was greater (*P* < 0.01) in diets containing XAP or protease compared with the control and in the diet containing XAP compared with the diet containing protease alone. Phosphorus retention was greater (*P* < 0.01) in the diets with XAP or protease alone compared with the control and in P10000 compared with XAP10000.

**Table 2.** Nutrient utilization (%) by broilers receiving diets supplemented with graded levels of enzymes containing protease activity alone or in combination with Xylanase and amylase activities.

Diet		1	2	3	4	5	6		Contrasts			
Description		NC	P5000	P10000	XAP2500	XAP5000	XAP10000	SEM	2 vs. 5	3 vs. 6	1 vs 2, 3	1 vs. 4, 5, 6
Ileal	DM	59.7	61.6	63.9	62.5	64.3	65.9	0.761	0.018	0.080	0.002	<0.001
	Nitrogen	69.7	72.0	74.4	73.3	76.2	76.5	0.811	0.001	0.076	0.001	<0.001
	EE	83.5	83.8	84.1	84.5	85.9	83.9	0.962	0.119	0.855	0.705	0.263
Total tract	DM	59.9	61.2	64.2	62.3	64.8	65.5	0.622	<0.001	0.145	0.001	<0.001
	Nitrogen	49.7	51.7	56.7	54.6	58.4	58.5	1.068	<0.001	0.245	0.002	<0.001
	AME, kcal/kg	2,415	2,453	2,592	2,482	2,622	2,597	26.4	<0.001	0.880	0.002	<0.001
	P	32.1	35.6	37.0	38.0	37.0	42.1	1.016	0.344	0.001	0.002	<0.001
	Ca	38.0	41.5	41.7	35.9	39.1	42.0	1.051	0.112	0.808	0.009	0.429
	Na	45.9	48.0	46.7	32.6	38.5	41.9	2.394	0.008	0.163	0.625	0.005

NC – negative control; P5000 – protease at 5,000 PU/kg; P10000 – protease at 10,000 PU/kg; XAP2500 – XAP with protease at 2,500 PU/kg; XAP5000 – XAP with protease at 5,000 PU/kg; XAP10000 – XAP with protease at 10,000 PU/kg.

DM – dry matter; EE – ether extract; GE – gross energy.

The pre-cecal flow of NSP components in response to the dietary treatments are shown in Table 3. Supplementation of protease or XAP reduced (*P* < 0.05) the pre-cecal flow of insoluble arabinose relative to the control but had no effect on other components. Supplementation of XAP alone reduced (*P* < 0.05) the pre-cecal flow of all NSP components except soluble xylose, soluble glucose, and soluble arabinose on which the enzyme had no effect. The pre-cecal flow of insoluble and total xylose, total glucose, as well as insoluble and total arabinose was lower (*P* < 0.05) in XAP5000 compared with P5000. However the pre-cecal flow of NSP components was not different between XAP10000 and P10000.

**Table 3.** Pre-cecal flow (g/100g dry matter intake) of components of non-starch polysaccharides in response to feeding diets supplemented with graded levels of enzymes containing protease activity alone or in combination with xylanase and amylase activities.

Diet number		1	2	3	4	5	6		Contrasts			
Description		NC	P5000	P10000	XAP2500	XAP5000	XAP10000	SEM	2 vs. 5	3 vs. 6	1 vs 2, 3	1 vs. 4, 5, 6
Xylose	Soluble	0.193	0.165	0.212	0.179	0.206	0.229	0.032	0.365	0.720	0.911	0.761
	Insoluble	2.50	2.51	2.28	2.37	2.23	2.14	0.056	0.001	0.086	0.137	<0.001
	Total	2.69	2.68	2.49	2.55	2.44	2.37	0.060	0.009	0.155	0.166	0.002
Glucose	Soluble	0.282	0.302	0.329	0.281	0.298	0.381	0.049	0.956	0.455	0.585	0.507
	Insoluble	2.90	2.87	2.66	2.72	2.62	2.45	0.087	0.051	0.090	0.207	0.004
	Total	3.18	3.17	2.99	3.00	2.92	2.83	0.075	0.023	0.137	0.268	0.004
Arabinose	Soluble	0.396	0.554	0.424	0.476	0.470	0.503	0.052	0.264	0.293	0.157	0.159
	Insoluble	2.35	2.28	2.09	2.14	2.05	1.94	0.060	0.011	0.073	0.030	<0.001
	Total	2.74	2.83	2.51	2.61	2.52	2.44	0.066	0.002	0.443	0.368	0.007
Galactose	Soluble	0.938	0.888	0.863	0.878	0.803	0.833	0.037	0.110	0.565	0.171	0.023
	Insoluble	1.55	1.56	1.41	1.39	1.44	1.27	0.069	0.239	0.157	0.441	0.028
	Total	2.49	2.45	2.27	2.27	2.25	2.10	0.084	0.099	0.161	0.221	0.006

NC – negative control; P5000 – protease at 5,000 PU/kg; P10000 – protease at 10,000 PU/kg; XAP2500 – XAP with protease at 2,500 PU/kg; XAP5000 – XAP with protease at 5,000 PU/kg; XAP10000 – XAP with protease at 10,000 PU/kg.

The data of post-cecal flow of NSP components are shown in Table 4. Diets supplemented with protease or XAP had lower (*P* < 0.05) post-cecal flow of total glucose, soluble galactose, and total galactose relative to the control; XAP supplementation reduced (*P* < 0.05) post-cecal flow of insoluble xylose, insoluble glucose, and insoluble galactose relative to the control diet. Treatment XAP5000 had lower (*P* < 0.05) post-cecal flow of insoluble and total glucose, as well as insoluble and total galactose compared with P5000. In addition, XAP10000 had lower (*P* < 0.05) post-cecal flow of insoluble glucose and insoluble galactose than P10000.

**Table 4.** Post-cecal flow (g/100g dry matter intake) of NSP components in response to feeding diets supplemented with graded levels of enzymes containing protease activity alone or in combination with xylanase and amylase activities.

Diet		1	2	3	4	5	6		Contrasts			
Description		NC	P5000	P 10000	XAP2500	XAP5000	XAP10000	SEM	2 vs. 5	3 vs. 6	1 vs. 2, 3	1 vs. 4, 5, 6
Xylose	Soluble	0.226	0.221	0.260	0.367	0.304	0.260	0.048	0.231	0.985	0.800	0.137
	Insoluble	2.53	2.48	1.93	2.27	2.16	2.12	0.138	0.117	0.343	0.064	0.038
	Total	2.75	2.70	2.19	2.64	2.47	2.38	0.145	0.267	0.370	0.091	0.129
Glucose	Soluble	0.198	0.187	0.249	0.430	0.216	0.274	0.056	0.712	0.760	0.773	0.099
	Insoluble	2.98	2.96	2.73	2.74	2.67	2.54	0.06	0.001	0.037	0.061	<.0001
	Total	3.24	3.15	2.96	3.17	2.88	2.82	0.06	0.005	0.127	0.023	0.000
Arabinose	Soluble	0.453	0.382	0.434	0.552	0.475	0.438	0.063	0.302	0.956	0.565	0.623
	Insoluble	2.11	2.12	1.58	1.89	1.84	1.77	0.123	0.121	0.298	0.099	0.063
	Total	2.56	2.50	2.01	2.44	2.32	2.20	0.146	0.380	0.364	0.098	0.164
Galactose	Soluble	0.770	0.675	0.624	0.730	0.660	0.667	0.035	0.760	0.404	0.009	0.044
	Insoluble	1.40	1.45	1.35	1.36	1.25	1.22	0.036	0.000	0.025	0.997	0.006
	Total	2.17	2.12	1.97	2.09	1.90	1.89	0.048	0.003	0.289	0.045	0.001

NC – negative control; P5000 – protease at 5,000 PU/kg; P10000 – protease at 10,000 PU/kg; XAP2500 – XAP with protease at 2,500 PU/kg; XAP5000 – XAP with protease at 5,000 PU/kg; XAP10000 – XAP with protease at 10,000 PU/kg.

The data on flow of the total NSP are shown in Table 5. Supplementation of XAP reduced (*P* < 0.01) pre-cecal flow of insoluble and total NSP relative to the control and protease supplementation decreased (*P* < 0.05) post-cecal flow of soluble NSP relative to the control. XAP5000 treatment had lower (*P* < 0.05) pre-cecal flow of insoluble and total NSP compared with P5000. The flow of RO (Table 6) was lower (*P* < 0.05) in diets supplemented with XAP compared with the control. In addition, pre-cecal and post-cecal flows of galactose and glucose in the RO, as well as the total RO were lower (*P* < 0.05) in diets supplemented with protease compared with the control. The pre-cecal flow of glucose in the RO fraction was lower (*P* < 0.05) in XAP5000 compared with P5000. The pre-cecal flow of galactose and total RO was lower (*P* ≤ 0.05) in XAP10000 compared with P10000 treatment.

**Table 5.** Pre- and post-cecal flows (g/100g dry matter intake) of non-starch polysaccharides in response to feeding diets supplemented with graded levels of enzymes containing protease activity alone or in combination with xylanase and amylase activities.

Diet		1	2	3	4	5	6		Contrasts			
Description		NC	P5000	P10000	XAP2500	XAP5000	XAP10000	SEM	2 vs. 5	3 vs. 6	1 vs. 2, 3	1 vs. 4, 5, 6
Pre-cecal	Soluble	2.73	2.73	2.64	2.70	2.54	2.75	0.138	0.337	0.594	0.789	0.669
	Insoluble	10.5	10.4	9.49	9.65	9.46	8.75	0.285	0.021	0.073	0.141	0.001
	Total	13.2	13.2	12.1	12.4	12.0	11.5	0.309	0.012	0.153	0.139	0.001
Post-cecal	Soluble	2.38	2.03	1.94	2.64	2.17	2.10	0.154	0.523	0.484	0.044	0.661
	Insoluble	10.2	10.2	7.94	9.35	8.93	8.67	0.549	0.122	0.355	0.107	0.071
	Total	12.5	12.2	9.88	12.0	11.1	10.8	0.635	0.231	0.332	0.061	0.095

NC – negative control; P5000 – protease at 5,000 PU/kg; P10000 – protease at 10,000 PU/kg; XAP2500 – XAP with protease at 2,500 PU/kg; XAP5000 – XAP with protease at 5,000 PU/kg; XAP10000 – XAP with protease at 10,000 PU/kg.

**Table 6.** Pre- and post-cecal flows (g/100g dry matter intake) of resistant oligosaccharides in broilers in response to feeding diets supplemented with graded levels of enzymes containing protease activity alone or in combination with xylanase and amylase activities.

Diet		1	2	3	4	5	6		Contrasts			
Description		NC	P5000	P10000	XAP250	XAP5000	XAP10000	SEM	2 vs. 5	3 vs. 6	1 vs 2, 3	1 vs. 4, 5, 6
Pre-cecal	Galactose	1.31	1.28	1.26	1.22	1.16	1.08	0.063	0.211	0.047	0.586	0.037
	Glucose	0.781	0.764	0.736	0.733	0.662	0.646	0.034	0.044	0.072	0.444	0.015
	Total	2.494	2.388	2.375	2.314	2.147	2.025	0.115	0.144	0.039	0.436	0.018
Post-cecal	Galactose	0.713	0.616	0.447	0.487	0.598	0.587	0.012	0.810	0.061	0.007	0.013
	Glucose	0.552	0.481	0.328	0.351	0.460	0.415	0.012	0.740	0.180	0.012	0.010
	Total	1.49	1.34	0.972	1.01	1.29	1.20	0.010	0.729	0.129	0.014	0.011

NC – negative control; P5000 – protease at 5,000 PU/kg; P10000 – protease at 10,000 PU/kg; XAP2500 – XAP with protease at 2,500 PU/kg; XAP5000 – XAP with protease at 5,000 PU/kg; XAP10000 – XAP with protease at 10,000 PU/kg.

## DISCUSSION

The objectives of the current study were to assess the ileal digestibility and total tract retention effects of protease without and with carbohydrases and the effects of the enzymes on the disappearance of NSP component sugars in 21-day-old broiler chickens fed a corn-based diet. Diets contained 10% corn-Distillers' Dried Grains with Soluble (DDGS), which increased the presence of total NSP compared with simple corn-soybean meal based diets. The experimental design allowed direct comparison of 2 doses of protease (5,000 or 10,000 PU/kg) versus enzyme combinations containing protease at the same doses, along with xylanase and amylase at fixed ratios with protease. An additional treatment tested the XAP combination at a lower dose, corresponding to 2,500 PU/kg.

The single subtilisin protease increased the apparent ileal digestibility of N particularly at the high enzyme dose (10,000 PU/kg), which agreed with previous reports with the use of mono-component alkaline proteases in broilers fed corn-based diets (Angel et al., 2011). Nitrogen digestibility was 4.2% points higher in diets containing XAP relative to diets with protease alone even when protease was at 5,000 PU/kg in both diets. At 10,000 PU/kg, N digestibility was 2.1% points higher with XAP compared with diets containing protease alone. This likely indicates a plateau of the effect of XAP on N digestibility between XAP5000 and XAP10000. Interestingly, N digestibility values in XAP5000 and P10000 were not different. It can be inferred from this that the effect of carbohydrases on top of protease was not additive after a certain threshold of protein digestibility. Recently, Romero et al. (2013, 2014) studied the apparent amino acid and protein digestibility effects of protease applied on top of carbohydrases in 21-day-old broilers, and reported increased protein digestibility as a result of the addition of protease, in contrast with more inconsistent effects of carbohydrases on protein digestibility. In the present study, the use of carbohydrases increased protein digestibility beyond that of protease only at a specific dose. These findings suggest that additivity of protein and amino acid matrices of proteases and carbohydrases cannot be generally assumed in diet formulation.

In the current study, a significant increment in the AME due to protease was evident, particularly at 10,000 PU/kg. Interestingly, no additivity of proteases and carbohydrases on energy utilization was evident at this dose, but this was evident at 5,000 PU/kg. Romero et al. (2013) reported marginal increments of AMEn with the addition of 5,000 PU/kg of the same protease on top of xylanase and amylase in corn-based diets fed to 21-day-old chickens, which was explained by the caloric content of increments of protein digestibility due to the enzyme. In another study, Romero et al. (2014) reported increments in AMEn with the addition of this protease on top of xylanase and amylase in corn- and wheat-based diets using 21-day-old and 42-day-old chickens, with greater increments in older birds, and in wheat-based diets, particularly with inclusion of corn DDGs and canola meal. In some particular diets of that study, the increments in energy digestibility with the addition of protease were not explained by increments in the digestibility of starch, fat, and protein; therefore, the authors suggested a possible effect on the ileal disappearance of NSP which was not measured in that study.

The current study measured the flow of individual sugars within the soluble and insoluble NSP fractions. The flow of NSP components is an indication of the concentration of NSP components in the dry matter. Logically, greater hydrolysis will reduce the amount of NSP components that is detected in the digesta or excreta and consequently lower values for NSP flow is an indication of greater NSP hydrolysis in the particular diet.

Generally NSP flows, pre- and post-cecal, were lower in diets with XAP compared with diets containing protease alone. This is an indication that combination of enzymes with carbohydrase and protease activities was more effective in reducing NSP flow than enzyme with protease activity alone. Others have similarly reported improved NSP degradation following the use of exogenous enzymes (Korcher et al., 2002; Ouhida et al., 2002; Barekatain et al., 2013). At the pre-cecal level, XAP was able to reduce the flow of insoluble xylose and arabinose, which are the most important components of hemicellulose in corn and corn-DDGS. This observation indicates an increase in the solubilization of arabinoxylan polymers in the small intestine. The xylanase used in the current study was an endo-1–4-xylanase of the GH11 family, which has been reported to cleave the xylose backbone of the arabinoxylan chain (Courtin and Delcour, 2002; Hu et al., 2008). Therefore, it would be expected that the presence of xylanase would reduce the flow of insoluble xylose, which would also bring arabinose substitutions into the soluble fraction.

There is close relationship between fiber and protein in corn (Rybka et al., 1992; Parker et al., 1999) and hence the use of protease by itself may have a limited effect on NSP hydrolysis by attacking the structural protein or glycoprotein as suggested by Cowieson (2010). The corn endosperm proteins are of 2 types, one of which is the matrix that also envelops the starch granules (Parker et al., 1999). Close fiber-protein interactions have been reported for cereals and oilseeds (Ouhida et al., 2002; Duodu et al., 2003). Interactions such as cross-linking is a physico-structural barrier to hydrolysis of structural and storage carbohydrates and the hydrolysis of the structural proteins by protease will in itself increase NSP hydrolysis. Consequently, it can be expected that the use of protease along with the carbohydrase will increase the efficacy of the carbohydrase as evidenced through decreased NSP component in the current experiment. It is interesting to note that the only NSP component which protease reduced its pre-cecal flow is insoluble arabinose, and this may be related to the ability of protease to increase access of the digestive enzyme to structural carbohydrate and thus reduce cellular integrity.

The XAP combination caused a reduction of the total flow of xylose and arabinose, which indicates disappearance, likely due to fermentation in the small intestine, which was not evident with the use of protease on its own. It appears that protease contributed to opening the tri-dimensional structure of hemicellulose, without a significant solubilization of arabinoxylo- oligosaccharides, for bacterial fermentation. Evidence for effects of xylanases in the solubilization and fermentation of fiber in the ceca of 21-day-old broiler chickens have been reported in corn and wheat based diets (Kiarie et al., 2014). However, reports on the disappearance of fiber in the small intestine are conflicting and have been limited to wheat based diets. Choct et al. (1999) reported that a combination of xylanase and protease reduced the production of short chain fatty acids (**SCFA**) in the ileum, but increased it in the cecum of 29-day-old broilers. In contrast, Wang et al. (2005) reported that a carbohydrase combination increased SCFA production in the ileum of broilers at 21 d, but had no effect at 42 d, whereas the production of SCFA in the cecum was increased at both 21 and 42 d. Logically, the activity of exogenous xylanases that are active in the gastrointestinal tract of chickens would be initiated as soon as the enzyme is released and moisture is sufficient to allow enzymatic activity, and would potentially solubilize hemicellulose in the small intestine.

The effect of protease alone on NSP flow was more apparent at the post-cecal level. This activity of the protease was likely indirect, perhaps involving activity of intestinal bacteria. Evidently, protease does not have the same ability as xylanase to solubilize xylose but appeared to allow a preferential solubilization of arabinose substituted side chains. It is established that microorganisms are both more abundant and of greater variety post-ileal (Gong et al., 2007; Yeoman et al., 2012) and hence it is reasonable to conclude that this contributes more to the hydrolysis of NSP observed post-ileal. There is the possibility that the action of microorganisms that are capable of hydrolyzing NSP, even if partially, will further enhance the ability of protease to hydrolyze structural carbohydrates that are, very likely, already partially weakened by microbial activity. In addition, at the post-cecal level, protease reduced the flow of many of NSP constituents, which may be the result of the joint action of both the protease and microbial activities.

Previous research using in-vitro and in-vivo ruminant models demonstrated the ability of exogenous subtilisin proteases to increase the solubilization and fermentation of hemicellulose (Colombatto and Beauchemin, 2009). The authors postulated that these enzymes acted by removing structural proteins in the cell wall, allowing faster access of ruminal microbes to digestible substrates. Taken as a whole, these results confirm the hypothesis of an indirect effect of this subtilisin protease in the solubilization and disappearance of different NSP sugar components. Those effects were certainly evident at the post-cecal level as confirmed by a significant effect in the disappearance of glucose and galactose from the NSP fraction. However as expected, effects on solubilization and the disappearance of NSP due to protease were not as vigorous compared with a combination of protease with xylanase and amylase. The experimental design did not allow a precise evaluation of additivity or synergy between these 3 enzymes, but certainly demonstrated that protease played a role in the solubilization and disappearance of fiber in this enzyme combination, which was enhanced when xylanase and amylase were present. Based on the extensive research of exogenous xylanase in poultry feed (Adeola and Cowieson, 2011; Slominski, 2011) and its biochemical activity, it can be inferred that xylanase, as opposed to amylase, was the major activity to produce these effects.

The most important RO in corn-soybean meal based diets are raffinose and stachyose, which together can make up to 5% of soybean meal (Choct et al., 2010). Additionally, β-mannans are also present in soy, but at lower concentrations compared to raffinose plus stachyose (Choct et al., 2010). Effects of enzymes in the flow of sugars from RO were analyzed with the understanding of the lack of specificity of the enzymes used to target the direct hydrolysis of bonds from these α-galactosides and from β-mannans. Protease reduced the flow of glucose and galactose from the RO fraction only post-cecal whereas XAP reduced the flow of these RO sugars in both pre- and post-cecal digesta. These effects were likely the result of fermentation resulting from the solubilization of the major fiber component of chicken feed, i.e., arabinoxylans from grains. These RO have been ascribed both prebiotic (Spring et al., 2000; Choct, 2006) as well as negative effects on gut health (Coon et al., 1990) depending on what responses have been measured in specific studies and what diets and animal models were used. The nature of these effects is likely dependent of the concentration of the specific RO as well as the health status of the flock. In any case, a reduction of the flow of RO through fermentation would probably have a neutral or beneficial effect in animal performance and gut health if it avoids negative effects of high concentrations of RO in the diet, or contributes to prebiotic effects of the same RO. What is not yet clear is the additional value of exogenous prebiotics in chicken diets, which naturally contain high levels of potentially prebiotic NSP, particularly when exogenous fibrolytic enzymes are used, some of which are capable of increasing the production of prebiotic oligosaccharides in-situ.

It can be concluded from the current study that whereas protease by itself improved nutrient utilization in corn-soybean meal diets and increased solubilization of NSP components, at the lower dose, a combination of xylanase, amylase, and protease produced effects greater than those of protease alone. However, the current data do not indicate an additivity in the effect of the enzymes when used in combination at higher doses.
